# {μ_2_-6,6′-Dimeth­oxy-2,2′-[butane-1,4-diylbis(nitrilo­methyl­idyne)]diphenolato}trinitratocopper(II)neodymium(III)

**DOI:** 10.1107/S1600536810040833

**Published:** 2010-10-20

**Authors:** Jing-Chun Xing, Xiao-Guang Cui, Bing Zhang, Wen-Zhi Li

**Affiliations:** a Department of Anesthesiology, the Second Affiliated Hospital, Harbin Medical University, Harbin 150081, People’s Republic of China

## Abstract

In the title complex, [CuNd(C_20_H_22_N_2_O_4_)(NO_3_)_3_], the Cu^II^ ion is coordinated in a distorted square-planar environment by two O atoms and two N atoms of a tetra­dentate Schiff base ligand. The Nd^III^ ion is ten-coordinated by three bis-chelating nitrate groups and four O atoms of the Schiff base ligand. The atoms of one of the nitrato ligands are disordered over two sets of sites, with refined occupancies of 0.567 (13) and 0.433 (17).

## Related literature

For the crystal structures of related copper–lanthanide complexes, see: Xing *et al.* (2008 ▶, 2009 ▶).
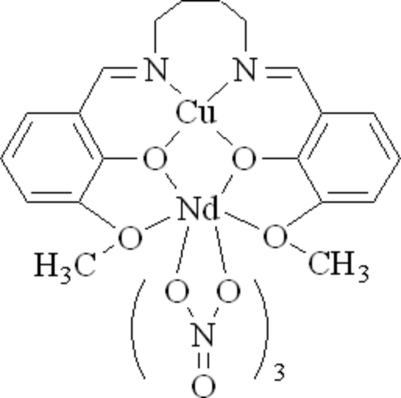


## Experimental

### 

#### Crystal data

[CuNd(C_20_H_22_N_2_O_4_)(NO_3_)_3_]
*M* *_r_* = 748.21
Monoclinic, 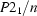

*a* = 11.729 (2) Å
*b* = 14.850 (3) Å
*c* = 15.063 (3) Å
β = 100.85 (3)°
*V* = 2576.7 (9) Å^3^
*Z* = 4
Mo *K*α radiation
μ = 2.89 mm^−1^
*T* = 295 K
0.22 × 0.18 × 0.13 mm

#### Data collection

Rigaku R-AXIS RAPID diffractometer
Absorption correction: multi-scan (*ABSCOR*; Higashi, 1995 ▶) *T*_min_ = 0.569, *T*_max_ = 0.705
24553 measured reflections
5867 independent reflections
4644 reflections with *I* > 2σ(*I*)
*R*_int_ = 0.045

#### Refinement

*R*[*F* ^2^ > 2σ(*F* ^2^)] = 0.042
*wR*(*F* ^2^) = 0.118
*S* = 1.06
5867 reflections
376 parameters
H-atom parameters constrained
Δρ_max_ = 1.32 e Å^−3^
Δρ_min_ = −1.79 e Å^−3^

### 

Data collection: *RAPID-AUTO* (Rigaku, 1998 ▶); cell refinement: *RAPID-AUTO*; data reduction: *CrystalStructure* (Rigaku/MSC, 2002 ▶); program(s) used to solve structure: *SHELXS97* (Sheldrick, 2008 ▶); program(s) used to refine structure: *SHELXL97* (Sheldrick, 2008 ▶); molecular graphics: *DIAMOND* (Brandenburg, 1999 ▶); software used to prepare material for publication: *SHELXL97*.

## Supplementary Material

Crystal structure: contains datablocks I. DOI: 10.1107/S1600536810040833/lh5135sup1.cif

Structure factors: contains datablocks I. DOI: 10.1107/S1600536810040833/lh5135Isup2.hkl

Additional supplementary materials:  crystallographic information; 3D view; checkCIF report

## Figures and Tables

**Fig. 1.**
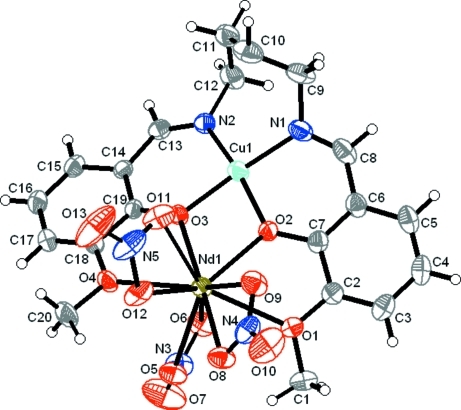
The molecular structure of the title complex, showing 40% probability displacement ellipsoids. All H atoms have been omitted for clarity. The disorder is not shown.

## supplementary crystallographic information

### Comment

The molecular structure of the title complex is shown in Fig.1. The octodentate Schiff base ligand links the Cu^II^ and Nd^III^ ions into a dinuclear complex through two phenolate O atoms, which is similar to the structures of copper-lanthanum complexes with similar ligands (Xing *et al.*, 2008;2009). The Nd^III^ ion is ten-coordinated by four oxygen atoms from the ligand and six oxygen atoms from three nitrate ions. The Cu^II^ ion is four-coordinated by two nitrogen atoms and two oxygen atoms from the ligand.

### Experimental

The title complex was obtained by the treatment of copper(II) acetate monohydrate (0.0499 g, 0.25 mmol) with the Schiff base (0.1595 g, 0.25 mmol) in methanol/acetone (20 ml:5 ml) at room temperature. Then the mixture was refluxed for 3 h after the addition of neodymium (III) nitrate hexahydrate (0.1595 g, 0.25 mmol). The reaction mixture was cooled and filtered; diethyl ether was allowed to diffuse slowly into the solution of the filtrate. Single crystals were obtained after several days. Analysis calculated for C_20_H_22_CuN_5_O_13_Nd: C, 32.08; H, 2.94; Cu, 8.49; N, 9.36; Nd, 19.28; found: C, 32.10; H, 2.98; Cu, 8.52; N, 9.39; Nd, 19.31%.

### Refinement

H atoms bound to C atoms were placed in calculated positions and treated as riding on their parent atoms, with C—H = 0.93 Å (aromatic), C—H = 0.97 Å (methylene), and with *U*_iso_(H) = 1.2*U*_eq_(C) or C—H = 0.96 Å (methyl) and with *U*_iso_(H) = 1.5*U*_eq_(C).

### Crystal data

**Table:**

[CuNd(C_20_H_22_N_2_O_4_)(NO_3_)_3_]	*F*(000) = 1480
*M**_r_* = 748.21	*D*_x_ = 1.929 Mg m^−^^3^
Monoclinic, *P*2_1_/*n*	Mo *K*α radiation, λ = 0.71073 Å
Hall symbol: -P 2yn	Cell parameters from 17594 reflections
*a* = 11.729 (2) Å	θ = 3.1–27.4°
*b* = 14.850 (3) Å	µ = 2.89 mm^−^^1^
*c* = 15.063 (3) Å	*T* = 295 K
β = 100.85 (3)°	Prism, brown
*V* = 2576.7 (9) Å^3^	0.22 × 0.18 × 0.13 mm
*Z* = 4	

### Data collection

**Table:**

Rigaku R-AXIS RAPID diffractometer	5867 independent reflections
Radiation source: fine-focus sealed tube	4644 reflections with *I* > 2σ(*I*)
graphite	*R*_int_ = 0.045
Detector resolution: 10.000 pixels mm^-1^	θ_max_ = 27.4°, θ_min_ = 3.1°
ω scans	*h* = −13→15
Absorption correction: multi-scan (*ABSCOR*;Higashi, 1995)	*k* = −19→19
*T*_min_ = 0.569, *T*_max_ = 0.705	*l* = −19→19
24553 measured reflections	

### Refinement

**Table:**

Refinement on *F*^2^	Primary atom site location: structure-invariant direct methods
Least-squares matrix: full	Secondary atom site location: difference Fourier map
*R*[*F*^2^ > 2σ(*F*^2^)] = 0.042	Hydrogen site location: inferred from neighbouring sites
*wR*(*F*^2^) = 0.118	H-atom parameters constrained
*S* = 1.06	*w* = 1/[σ^2^(*F*_o_^2^) + (0.0577*P*)^2^ + 4.6815*P*] where *P* = (*F*_o_^2^ + 2*F*_c_^2^)/3
5867 reflections	(Δ/σ)_max_ = 0.001
376 parameters	Δρ_max_ = 1.32 e Å^−^^3^
0 restraints	Δρ_min_ = −1.79 e Å^−^^3^

### Special details

**Table:**

Geometry. All e.s.d.'s (except the e.s.d. in the dihedral angle between two l.s. planes) are estimated using the full covariance matrix. The cell e.s.d.'s are taken into account individually in the estimation of e.s.d.'s in distances, angles and torsion angles; correlations between e.s.d.'s in cell parameters are only used when they are defined by crystal symmetry. An approximate (isotropic) treatment of cell e.s.d.'s is used for estimating e.s.d.'s involving l.s. planes.
Refinement. Refinement of *F*^2^ against ALL reflections. The weighted *R*-factor *wR* and goodness of fit *S* are based on *F*^2^, conventional *R*-factors *R* are based on *F*, with *F* set to zero for negative *F*^2^. The threshold expression of *F*^2^ > σ(*F*^2^) is used only for calculating *R*-factors(gt) *etc*. and is not relevant to the choice of reflections for refinement. *R*-factors based on *F*^2^ are statistically about twice as large as those based on *F*, and *R*- factors based on ALL data will be even larger.

### Fractional atomic coordinates and isotropic or equivalent isotropic displacement parameters (Å^2^)

**Table:**

	*x*	*y*	*z*	*U*_iso_*/*U*_eq_	Occ. (<1)
Nd1	0.25894 (2)	0.250636 (15)	0.565858 (17)	0.03466 (10)	
Cu1	0.55090 (5)	0.26892 (4)	0.53616 (4)	0.04027 (16)	
O1	0.2027 (3)	0.1636 (2)	0.4114 (2)	0.0475 (8)	
O2	0.4131 (3)	0.1970 (2)	0.4958 (2)	0.0439 (8)	
O3	0.4360 (3)	0.3442 (2)	0.5784 (2)	0.0366 (7)	
O4	0.2599 (3)	0.4087 (2)	0.6418 (2)	0.0445 (8)	
O5	0.0721 (3)	0.3308 (2)	0.5050 (3)	0.0517 (9)	
O6	0.2129 (3)	0.3654 (3)	0.4382 (3)	0.0529 (9)	
O7	0.0552 (5)	0.4417 (4)	0.4096 (4)	0.105 (2)	
O8	0.0829 (3)	0.1495 (3)	0.5693 (3)	0.0550 (9)	
O9	0.2512 (3)	0.0858 (2)	0.5984 (3)	0.0564 (10)	
O10	0.0964 (5)	0.0073 (3)	0.6032 (4)	0.0943 (17)	
N5'	0.342 (3)	0.2126 (16)	0.761 (2)	0.069 (6)	0.433 (13)
O11'	0.410 (2)	0.2061 (16)	0.7055 (19)	0.069 (4)	0.433 (13)
O12'	0.2416 (15)	0.240 (2)	0.7292 (19)	0.059 (3)	0.433 (13)
O13'	0.373 (2)	0.1966 (15)	0.8385 (13)	0.121 (7)	0.433 (13)
N5	0.304 (2)	0.2457 (12)	0.7648 (17)	0.069 (6)	0.567 (13)
O11	0.3847 (17)	0.2374 (13)	0.7216 (13)	0.069 (4)	0.567 (13)
O12	0.1995 (11)	0.2380 (14)	0.7202 (13)	0.059 (3)	0.567 (13)
O13	0.3259 (19)	0.2521 (11)	0.8489 (9)	0.121 (7)	0.567 (13)
N1	0.6498 (4)	0.1632 (3)	0.5145 (3)	0.0495 (11)	
N2	0.6655 (3)	0.3664 (3)	0.5549 (3)	0.0366 (8)	
N3	0.1118 (4)	0.3809 (3)	0.4494 (3)	0.0547 (12)	
N4	0.1408 (4)	0.0791 (3)	0.5906 (3)	0.0542 (11)	
C1	0.0887 (5)	0.1565 (5)	0.3578 (5)	0.0682 (18)	
H1A	0.0907	0.1732	0.2966	0.102*	
H1B	0.0370	0.1960	0.3816	0.102*	
H1C	0.0617	0.0956	0.3593	0.102*	
C2	0.2896 (4)	0.1137 (3)	0.3844 (3)	0.0401 (10)	
C3	0.2723 (5)	0.0496 (4)	0.3175 (4)	0.0524 (13)	
H3A	0.1977	0.0380	0.2859	0.063*	
C4	0.3658 (6)	0.0023 (4)	0.2970 (4)	0.0598 (15)	
H4A	0.3536	−0.0410	0.2516	0.072*	
C5	0.4759 (5)	0.0187 (4)	0.3430 (4)	0.0535 (14)	
H5A	0.5378	−0.0148	0.3302	0.064*	
C6	0.4959 (4)	0.0866 (3)	0.4103 (4)	0.0443 (11)	
C7	0.4019 (4)	0.1341 (3)	0.4317 (3)	0.0384 (10)	
C8	0.6123 (5)	0.0994 (3)	0.4591 (4)	0.0516 (13)	
H8A	0.6666	0.0566	0.4495	0.062*	
C9	0.7734 (5)	0.1518 (4)	0.5613 (5)	0.0608 (16)	
H9A	0.7854	0.0899	0.5815	0.073*	
H9B	0.8238	0.1633	0.5184	0.073*	
C10	0.8068 (5)	0.2126 (5)	0.6401 (4)	0.0622 (16)	
H10A	0.8639	0.1824	0.6853	0.075*	
H10B	0.7390	0.2242	0.6666	0.075*	
C11	0.8565 (5)	0.3017 (4)	0.6162 (4)	0.0594 (15)	
H11A	0.8647	0.3415	0.6681	0.071*	
H11B	0.9332	0.2915	0.6030	0.071*	
C12	0.7822 (4)	0.3475 (4)	0.5360 (4)	0.0507 (13)	
H12A	0.8186	0.4034	0.5232	0.061*	
H12B	0.7755	0.3090	0.4833	0.061*	
C13	0.6470 (4)	0.4465 (3)	0.5813 (3)	0.0366 (10)	
H13A	0.7072	0.4875	0.5824	0.044*	
C14	0.5442 (4)	0.4804 (3)	0.6095 (3)	0.0316 (9)	
C15	0.5476 (4)	0.5709 (3)	0.6402 (3)	0.0418 (11)	
H15A	0.6131	0.6061	0.6396	0.050*	
C16	0.4541 (4)	0.6062 (3)	0.6707 (4)	0.0459 (12)	
H16A	0.4559	0.6657	0.6904	0.055*	
C17	0.3572 (4)	0.5538 (3)	0.6725 (3)	0.0440 (11)	
H17A	0.2946	0.5779	0.6942	0.053*	
C18	0.3526 (4)	0.4661 (3)	0.6423 (3)	0.0347 (9)	
C19	0.4461 (4)	0.4276 (3)	0.6092 (3)	0.0301 (9)	
C20	0.1685 (5)	0.4403 (4)	0.6868 (5)	0.0643 (17)	
H20A	0.1998	0.4508	0.7495	0.097*	
H20B	0.1084	0.3957	0.6815	0.097*	
H20C	0.1368	0.4954	0.6592	0.097*	

### Atomic displacement parameters (Å^2^)

**Table:**

	*U*^11^	*U*^22^	*U*^33^	*U*^12^	*U*^13^	*U*^23^
Nd1	0.03264 (15)	0.03176 (15)	0.04069 (16)	−0.00236 (9)	0.00975 (11)	0.00024 (9)
Cu1	0.0327 (3)	0.0394 (3)	0.0499 (4)	−0.0008 (2)	0.0108 (3)	−0.0036 (3)
O1	0.0373 (18)	0.051 (2)	0.051 (2)	−0.0014 (16)	0.0001 (15)	−0.0091 (17)
O2	0.0324 (16)	0.0447 (18)	0.056 (2)	−0.0078 (14)	0.0118 (15)	−0.0193 (16)
O3	0.0317 (16)	0.0320 (15)	0.0488 (18)	−0.0047 (13)	0.0146 (14)	−0.0081 (14)
O4	0.0328 (17)	0.0444 (18)	0.061 (2)	−0.0023 (14)	0.0216 (16)	−0.0115 (16)
O5	0.0390 (19)	0.055 (2)	0.063 (2)	0.0002 (16)	0.0136 (17)	0.0114 (18)
O6	0.047 (2)	0.055 (2)	0.061 (2)	−0.0024 (17)	0.0220 (18)	0.0114 (18)
O7	0.082 (3)	0.092 (4)	0.142 (5)	0.032 (3)	0.023 (3)	0.067 (4)
O8	0.0376 (19)	0.052 (2)	0.075 (3)	−0.0042 (17)	0.0098 (18)	0.007 (2)
O9	0.054 (2)	0.0395 (19)	0.077 (3)	0.0041 (17)	0.015 (2)	0.0063 (18)
O10	0.115 (4)	0.054 (3)	0.109 (4)	−0.043 (3)	0.009 (3)	0.012 (3)
N5'	0.086 (15)	0.067 (12)	0.049 (4)	−0.047 (9)	−0.003 (8)	0.014 (9)
O11'	0.059 (9)	0.086 (12)	0.058 (8)	−0.019 (7)	−0.001 (5)	0.008 (7)
O12'	0.048 (8)	0.075 (3)	0.058 (5)	−0.044 (8)	0.018 (8)	−0.005 (3)
O13'	0.171 (15)	0.140 (13)	0.043 (4)	−0.087 (12)	0.000 (6)	0.006 (8)
N5	0.086 (15)	0.067 (12)	0.049 (4)	−0.047 (9)	−0.003 (8)	0.014 (9)
O11	0.059 (9)	0.086 (12)	0.058 (8)	−0.019 (7)	−0.001 (5)	0.008 (7)
O12	0.048 (8)	0.075 (3)	0.058 (5)	−0.044 (8)	0.018 (8)	−0.005 (3)
O13	0.171 (15)	0.140 (13)	0.043 (4)	−0.087 (12)	0.000 (6)	0.006 (8)
N1	0.035 (2)	0.049 (2)	0.067 (3)	0.0028 (19)	0.015 (2)	−0.001 (2)
N2	0.0322 (19)	0.038 (2)	0.042 (2)	−0.0054 (16)	0.0115 (16)	−0.0019 (16)
N3	0.051 (3)	0.045 (2)	0.066 (3)	0.002 (2)	0.006 (2)	0.013 (2)
N4	0.067 (3)	0.041 (2)	0.054 (3)	−0.019 (2)	0.009 (2)	0.000 (2)
C1	0.038 (3)	0.086 (5)	0.073 (4)	0.000 (3)	−0.007 (3)	−0.017 (4)
C2	0.041 (3)	0.042 (3)	0.039 (2)	−0.003 (2)	0.010 (2)	0.002 (2)
C3	0.063 (3)	0.053 (3)	0.040 (3)	−0.010 (3)	0.007 (2)	−0.006 (2)
C4	0.080 (4)	0.053 (3)	0.049 (3)	0.000 (3)	0.020 (3)	−0.016 (3)
C5	0.066 (4)	0.047 (3)	0.056 (3)	0.004 (3)	0.031 (3)	−0.006 (2)
C6	0.046 (3)	0.042 (3)	0.050 (3)	−0.004 (2)	0.022 (2)	−0.004 (2)
C7	0.042 (3)	0.034 (2)	0.041 (2)	−0.0062 (19)	0.012 (2)	−0.0018 (19)
C8	0.048 (3)	0.039 (3)	0.075 (4)	0.003 (2)	0.031 (3)	0.002 (3)
C9	0.034 (3)	0.051 (3)	0.095 (5)	0.008 (2)	0.008 (3)	0.009 (3)
C10	0.047 (3)	0.080 (4)	0.059 (4)	0.018 (3)	0.009 (3)	0.019 (3)
C11	0.037 (3)	0.063 (4)	0.080 (4)	−0.004 (3)	0.018 (3)	−0.011 (3)
C12	0.043 (3)	0.046 (3)	0.070 (4)	−0.008 (2)	0.030 (3)	−0.002 (3)
C13	0.034 (2)	0.039 (2)	0.037 (2)	−0.0070 (19)	0.0059 (19)	0.0032 (19)
C14	0.030 (2)	0.035 (2)	0.030 (2)	−0.0031 (17)	0.0043 (17)	−0.0025 (17)
C15	0.045 (3)	0.036 (2)	0.043 (3)	−0.007 (2)	0.003 (2)	−0.006 (2)
C16	0.049 (3)	0.036 (2)	0.053 (3)	−0.003 (2)	0.010 (2)	−0.013 (2)
C17	0.043 (3)	0.042 (3)	0.048 (3)	0.006 (2)	0.011 (2)	−0.010 (2)
C18	0.033 (2)	0.034 (2)	0.038 (2)	0.0009 (18)	0.0086 (18)	−0.0026 (18)
C19	0.033 (2)	0.028 (2)	0.029 (2)	0.0001 (17)	0.0047 (17)	−0.0024 (16)
C20	0.044 (3)	0.072 (4)	0.087 (5)	−0.003 (3)	0.038 (3)	−0.024 (3)

### Geometric parameters (Å, °)

**Table:**

Nd1—O2	2.395 (3)	C1—H1B	0.9600
Nd1—O3	2.476 (3)	C1—H1C	0.9600
Nd1—O9	2.501 (4)	C2—C3	1.373 (7)
Nd1—O5	2.510 (4)	C2—C7	1.408 (7)
Nd1—O12'	2.51 (3)	C3—C4	1.386 (8)
Nd1—O11	2.53 (2)	C3—H3A	0.9300
Nd1—O6	2.550 (4)	C4—C5	1.368 (8)
Nd1—O12	2.557 (19)	C4—H4A	0.9300
Nd1—O8	2.562 (4)	C5—C6	1.418 (7)
Nd1—O11'	2.57 (3)	C5—H5A	0.9300
Nd1—O4	2.609 (3)	C6—C7	1.397 (7)
Nd1—O1	2.634 (3)	C6—C8	1.436 (7)
Cu1—O2	1.938 (3)	C8—H8A	0.9300
Cu1—O3	1.949 (3)	C9—C10	1.484 (9)
Cu1—N2	1.959 (4)	C9—H9A	0.9700
Cu1—N1	2.015 (4)	C9—H9B	0.9700
O1—C2	1.383 (6)	C10—C11	1.517 (9)
O1—C1	1.431 (6)	C10—H10A	0.9700
O2—C7	1.332 (5)	C10—H10B	0.9700
O3—C19	1.319 (5)	C11—C12	1.511 (8)
O4—C18	1.381 (5)	C11—H11A	0.9700
O4—C20	1.451 (6)	C11—H11B	0.9700
O5—N3	1.272 (6)	C12—H12A	0.9700
O6—N3	1.250 (6)	C12—H12B	0.9700
O7—N3	1.211 (6)	C13—C14	1.442 (6)
O8—N4	1.256 (6)	C13—H13A	0.9300
O9—N4	1.283 (6)	C14—C19	1.392 (6)
O10—N4	1.217 (6)	C14—C15	1.418 (6)
N5'—O13'	1.18 (4)	C15—C16	1.371 (7)
N5'—O12'	1.25 (4)	C15—H15A	0.9300
N5'—O11'	1.26 (5)	C16—C17	1.382 (7)
N5—O13	1.25 (3)	C16—H16A	0.9300
N5—O11	1.25 (3)	C17—C18	1.376 (6)
N5—O12	1.29 (2)	C17—H17A	0.9300
N1—C8	1.284 (7)	C18—C19	1.409 (6)
N1—C9	1.498 (6)	C20—H20A	0.9600
N2—C13	1.286 (6)	C20—H20B	0.9600
N2—C12	1.476 (6)	C20—H20C	0.9600
C1—H1A	0.9600		
			
O2—Nd1—O3	61.89 (10)	O11—N5—O12	117 (2)
O2—Nd1—O9	79.58 (13)	O13—N5—Nd1	174.0 (13)
O3—Nd1—O9	126.52 (12)	O11—N5—Nd1	58.8 (14)
O2—Nd1—O5	132.55 (12)	O12—N5—Nd1	60.0 (13)
O3—Nd1—O5	115.05 (11)	N5—O11—Nd1	96.2 (15)
O9—Nd1—O5	118.32 (12)	N5—O12—Nd1	94.1 (14)
O2—Nd1—O12'	128.2 (6)	C8—N1—C9	113.9 (5)
O3—Nd1—O12'	100.5 (4)	C8—N1—Cu1	122.9 (4)
O9—Nd1—O12'	74.5 (7)	C9—N1—Cu1	123.2 (4)
O5—Nd1—O12'	99.3 (6)	C13—N2—C12	116.9 (4)
O2—Nd1—O11	91.5 (4)	C13—N2—Cu1	125.3 (3)
O3—Nd1—O11	68.8 (5)	C12—N2—Cu1	117.9 (3)
O9—Nd1—O11	77.2 (5)	O7—N3—O6	121.1 (5)
O5—Nd1—O11	133.7 (4)	O7—N3—O5	121.8 (5)
O12'—Nd1—O11	39.5 (6)	O6—N3—O5	117.1 (4)
O2—Nd1—O6	87.72 (12)	O7—N3—Nd1	172.4 (5)
O3—Nd1—O6	74.83 (12)	O6—N3—Nd1	59.6 (2)
O9—Nd1—O6	142.32 (14)	O5—N3—Nd1	57.9 (2)
O5—Nd1—O6	50.31 (12)	O10—N4—O8	122.9 (5)
O12'—Nd1—O6	137.4 (7)	O10—N4—O9	120.6 (5)
O11—Nd1—O6	138.9 (5)	O8—N4—O9	116.5 (4)
O2—Nd1—O12	137.2 (4)	O10—N4—Nd1	177.3 (4)
O3—Nd1—O12	109.9 (3)	O8—N4—Nd1	59.6 (2)
O9—Nd1—O12	74.0 (5)	O9—N4—Nd1	57.0 (2)
O5—Nd1—O12	89.9 (4)	O1—C1—H1A	109.5
O12'—Nd1—O12	11.0 (5)	O1—C1—H1B	109.5
O11—Nd1—O12	50.4 (5)	H1A—C1—H1B	109.5
O6—Nd1—O12	132.8 (5)	O1—C1—H1C	109.5
O2—Nd1—O8	119.49 (12)	H1A—C1—H1C	109.5
O3—Nd1—O8	174.30 (12)	H1B—C1—H1C	109.5
O9—Nd1—O8	50.46 (12)	C3—C2—O1	124.9 (5)
O5—Nd1—O8	68.53 (12)	C3—C2—C7	121.1 (5)
O12'—Nd1—O8	74.3 (4)	O1—C2—C7	114.0 (4)
O11—Nd1—O8	105.5 (5)	C2—C3—C4	120.1 (5)
O6—Nd1—O8	110.47 (12)	C2—C3—H3A	120.0
O12—Nd1—O8	65.2 (3)	C4—C3—H3A	120.0
O2—Nd1—O11'	79.3 (5)	C5—C4—C3	120.5 (5)
O3—Nd1—O11'	69.1 (7)	C5—C4—H4A	119.7
O9—Nd1—O11'	68.4 (6)	C3—C4—H4A	119.7
O5—Nd1—O11'	147.2 (5)	C4—C5—C6	120.2 (5)
O12'—Nd1—O11'	49.6 (7)	C4—C5—H5A	119.9
O11—Nd1—O11'	14.0 (5)	C6—C5—H5A	119.9
O6—Nd1—O11'	143.7 (7)	C7—C6—C5	119.5 (5)
O12—Nd1—O11'	60.0 (6)	C7—C6—C8	122.1 (5)
O8—Nd1—O11'	105.5 (7)	C5—C6—C8	118.3 (5)
O2—Nd1—O4	123.43 (10)	O2—C7—C6	123.2 (4)
O3—Nd1—O4	61.78 (10)	O2—C7—C2	118.2 (4)
O9—Nd1—O4	142.31 (13)	C6—C7—C2	118.6 (4)
O5—Nd1—O4	70.42 (12)	N1—C8—C6	127.3 (5)
O12'—Nd1—O4	67.8 (7)	N1—C8—H8A	116.3
O11—Nd1—O4	73.1 (4)	C6—C8—H8A	116.3
O6—Nd1—O4	73.29 (12)	C10—C9—N1	113.2 (5)
O12—Nd1—O4	69.3 (5)	C10—C9—H9A	108.9
O8—Nd1—O4	117.08 (11)	N1—C9—H9A	108.9
O11'—Nd1—O4	85.8 (5)	C10—C9—H9B	108.9
O2—Nd1—O1	62.08 (11)	N1—C9—H9B	108.9
O3—Nd1—O1	114.20 (11)	H9A—C9—H9B	107.7
O9—Nd1—O1	71.39 (13)	C9—C10—C11	113.3 (5)
O5—Nd1—O1	81.61 (12)	C9—C10—H10A	108.9
O12'—Nd1—O1	141.4 (6)	C11—C10—H10A	108.9
O11—Nd1—O1	141.7 (4)	C9—C10—H10B	108.9
O6—Nd1—O1	71.31 (12)	C11—C10—H10B	108.9
O12—Nd1—O1	134.5 (4)	H10A—C10—H10B	107.7
O8—Nd1—O1	70.22 (13)	C12—C11—C10	113.1 (5)
O11'—Nd1—O1	128.0 (4)	C12—C11—H11A	109.0
O4—Nd1—O1	143.89 (12)	C10—C11—H11A	109.0
O2—Cu1—O3	80.28 (13)	C12—C11—H11B	109.0
O2—Cu1—N2	164.12 (16)	C10—C11—H11B	109.0
O3—Cu1—N2	91.55 (14)	H11A—C11—H11B	107.8
O2—Cu1—N1	89.51 (16)	N2—C12—C11	110.3 (4)
O3—Cu1—N1	162.74 (16)	N2—C12—H12A	109.6
N2—Cu1—N1	101.46 (17)	C11—C12—H12A	109.6
O2—Cu1—Nd1	39.55 (9)	N2—C12—H12B	109.6
O3—Cu1—Nd1	42.17 (9)	C11—C12—H12B	109.6
N2—Cu1—Nd1	133.71 (11)	H12A—C12—H12B	108.1
N1—Cu1—Nd1	124.04 (12)	N2—C13—C14	127.8 (4)
C2—O1—C1	117.2 (4)	N2—C13—H13A	116.1
C2—O1—Nd1	116.6 (3)	C14—C13—H13A	116.1
C1—O1—Nd1	125.9 (3)	C19—C14—C15	120.5 (4)
C7—O2—Cu1	125.2 (3)	C19—C14—C13	122.6 (4)
C7—O2—Nd1	125.0 (3)	C15—C14—C13	116.9 (4)
Cu1—O2—Nd1	109.44 (14)	C16—C15—C14	119.8 (4)
C19—O3—Cu1	129.1 (3)	C16—C15—H15A	120.1
C19—O3—Nd1	125.0 (3)	C14—C15—H15A	120.1
Cu1—O3—Nd1	105.93 (12)	C15—C16—C17	120.2 (4)
C18—O4—C20	116.6 (4)	C15—C16—H16A	119.9
C18—O4—Nd1	119.8 (2)	C17—C16—H16A	119.9
C20—O4—Nd1	123.6 (3)	C18—C17—C16	120.5 (5)
N3—O5—Nd1	96.6 (3)	C18—C17—H17A	119.7
N3—O6—Nd1	95.3 (3)	C16—C17—H17A	119.7
N4—O8—Nd1	95.4 (3)	C17—C18—O4	124.6 (4)
N4—O9—Nd1	97.6 (3)	C17—C18—C19	121.1 (4)
O13'—N5'—O12'	123 (3)	O4—C18—C19	114.3 (4)
O13'—N5'—O11'	121 (3)	O3—C19—C14	123.4 (4)
O12'—N5'—O11'	116 (3)	O3—C19—C18	118.7 (4)
O13'—N5'—Nd1	179 (3)	C14—C19—C18	117.9 (4)
O12'—N5'—Nd1	56.6 (18)	O4—C20—H20A	109.5
O11'—N5'—Nd1	59.2 (18)	O4—C20—H20B	109.5
N5'—O11'—Nd1	95.8 (19)	H20A—C20—H20B	109.5
N5'—O12'—Nd1	99 (2)	O4—C20—H20C	109.5
O13—N5—O11	120 (2)	H20A—C20—H20C	109.5
O13—N5—O12	122 (2)	H20B—C20—H20C	109.5
			
O3—Nd1—Cu1—O2	−160.4 (2)	O6—Nd1—N5'—O11'	−97.7 (17)
O9—Nd1—Cu1—O2	49.8 (2)	O12—Nd1—N5'—O11'	176 (2)
O5—Nd1—Cu1—O2	−100.2 (2)	O8—Nd1—N5'—O11'	131.7 (17)
O12'—Nd1—Cu1—O2	127.1 (8)	O4—Nd1—N5'—O11'	−110.1 (17)
O11—Nd1—Cu1—O2	123.2 (5)	O1—Nd1—N5'—O11'	74 (2)
O6—Nd1—Cu1—O2	−92.6 (2)	O13'—N5'—O11'—Nd1	179 (2)
O12—Nd1—Cu1—O2	127.0 (6)	O12'—N5'—O11'—Nd1	−3(3)
O8—Nd1—Cu1—O2	30.4 (2)	O2—Nd1—O11'—N5'	−168.7 (17)
O11'—Nd1—Cu1—O2	110.6 (6)	O3—Nd1—O11'—N5'	127.5 (17)
O4—Nd1—Cu1—O2	−165.54 (19)	O9—Nd1—O11'—N5'	−85.7 (17)
O1—Nd1—Cu1—O2	−21.42 (19)	O5—Nd1—O11'—N5'	23 (2)
O2—Nd1—Cu1—O3	160.4 (2)	O12'—Nd1—O11'—N5'	2.0 (15)
O9—Nd1—Cu1—O3	−149.77 (18)	O11—Nd1—O11'—N5'	41 (4)
O5—Nd1—Cu1—O3	60.23 (19)	O6—Nd1—O11'—N5'	120.3 (18)
O12'—Nd1—Cu1—O3	−72.5 (8)	O12—Nd1—O11'—N5'	−2.3 (16)
O11—Nd1—Cu1—O3	−76.4 (4)	O8—Nd1—O11'—N5'	−50.8 (17)
O6—Nd1—Cu1—O3	67.80 (17)	O4—Nd1—O11'—N5'	66.2 (16)
O12—Nd1—Cu1—O3	−72.6 (6)	O1—Nd1—O11'—N5'	−127.5 (14)
O8—Nd1—Cu1—O3	−169.2 (2)	O13'—N5'—O12'—Nd1	−179 (2)
O11'—Nd1—Cu1—O3	−89.0 (6)	O11'—N5'—O12'—Nd1	3(3)
O4—Nd1—Cu1—O3	−5.14 (17)	O2—Nd1—O12'—N5'	10 (2)
O1—Nd1—Cu1—O3	138.99 (17)	O3—Nd1—O12'—N5'	−52.7 (18)
O2—Nd1—Cu1—N2	158.8 (2)	O9—Nd1—O12'—N5'	72.5 (17)
O3—Nd1—Cu1—N2	−1.6 (2)	O5—Nd1—O12'—N5'	−170.5 (17)
O9—Nd1—Cu1—N2	−151.41 (19)	O11—Nd1—O12'—N5'	−16.0 (14)
O5—Nd1—Cu1—N2	58.6 (2)	O6—Nd1—O12'—N5'	−131.6 (16)
O12'—Nd1—Cu1—N2	−74.1 (8)	O12—Nd1—O12'—N5'	158 (7)
O11—Nd1—Cu1—N2	−78.0 (5)	O8—Nd1—O12'—N5'	125.1 (18)
O6—Nd1—Cu1—N2	66.16 (19)	O11'—Nd1—O12'—N5'	−2.0 (16)
O12—Nd1—Cu1—N2	−74.2 (6)	O4—Nd1—O12'—N5'	−106.2 (17)
O8—Nd1—Cu1—N2	−170.9 (2)	O1—Nd1—O12'—N5'	101.3 (16)
O11'—Nd1—Cu1—N2	−90.6 (6)	O2—Nd1—N5—O11	15.0 (14)
O4—Nd1—Cu1—N2	−6.77 (18)	O3—Nd1—N5—O11	−41.8 (13)
O1—Nd1—Cu1—N2	137.35 (18)	O9—Nd1—N5—O11	86.7 (12)
O2—Nd1—Cu1—N1	−33.5 (2)	O5—Nd1—N5—O11	−157.5 (12)
O3—Nd1—Cu1—N1	166.1 (2)	O12'—Nd1—N5—O11	164 (3)
O9—Nd1—Cu1—N1	16.4 (2)	O6—Nd1—N5—O11	−104.4 (12)
O5—Nd1—Cu1—N1	−133.6 (2)	O12—Nd1—N5—O11	166 (2)
O12'—Nd1—Cu1—N1	93.6 (8)	O8—Nd1—N5—O11	136.7 (13)
O11—Nd1—Cu1—N1	89.8 (5)	O11'—Nd1—N5—O11	16.1 (19)
O6—Nd1—Cu1—N1	−126.06 (19)	O4—Nd1—N5—O11	−102.1 (14)
O12—Nd1—Cu1—N1	93.6 (6)	O1—Nd1—N5—O11	93.0 (17)
O8—Nd1—Cu1—N1	−3.1 (2)	O2—Nd1—N5—O12	−150.9 (13)
O11'—Nd1—Cu1—N1	77.2 (6)	O3—Nd1—N5—O12	152.4 (13)
O4—Nd1—Cu1—N1	161.00 (18)	O9—Nd1—N5—O12	−79.1 (13)
O1—Nd1—Cu1—N1	−54.87 (19)	O5—Nd1—N5—O12	36.7 (14)
O2—Nd1—O1—C2	16.3 (3)	O12'—Nd1—N5—O12	−2(4)
O3—Nd1—O1—C2	51.0 (3)	O11—Nd1—N5—O12	−166 (2)
O9—Nd1—O1—C2	−71.5 (3)	O6—Nd1—N5—O12	89.8 (14)
O5—Nd1—O1—C2	164.7 (3)	O8—Nd1—N5—O12	−29.1 (13)
O12'—Nd1—O1—C2	−100.9 (10)	O11'—Nd1—N5—O12	−149.7 (18)
O11—Nd1—O1—C2	−35.0 (9)	O4—Nd1—N5—O12	92.1 (14)
O6—Nd1—O1—C2	113.9 (3)	O1—Nd1—N5—O12	−72.8 (16)
O12—Nd1—O1—C2	−113.8 (7)	O13—N5—O11—Nd1	−173.1 (14)
O8—Nd1—O1—C2	−125.2 (3)	O12—N5—O11—Nd1	14 (2)
O11'—Nd1—O1—C2	−30.7 (9)	O2—Nd1—O11—N5	−166.6 (12)
O4—Nd1—O1—C2	125.7 (3)	O3—Nd1—O11—N5	134.5 (13)
O2—Nd1—O1—C1	−170.0 (5)	O9—Nd1—O11—N5	−87.6 (13)
O3—Nd1—O1—C1	−135.4 (4)	O5—Nd1—O11—N5	29.5 (16)
O9—Nd1—O1—C1	102.1 (5)	O12'—Nd1—O11—N5	−6.5 (14)
O5—Nd1—O1—C1	−21.6 (5)	O6—Nd1—O11—N5	105.2 (14)
O12'—Nd1—O1—C1	72.8 (10)	O12—Nd1—O11—N5	−8.0 (12)
O11—Nd1—O1—C1	138.6 (9)	O8—Nd1—O11—N5	−45.3 (13)
O6—Nd1—O1—C1	−72.5 (5)	O11'—Nd1—O11—N5	−137 (6)
O12—Nd1—O1—C1	59.9 (8)	O4—Nd1—O11—N5	68.8 (12)
O8—Nd1—O1—C1	48.5 (4)	O1—Nd1—O11—N5	−122.9 (10)
O11'—Nd1—O1—C1	143.0 (9)	O13—N5—O12—Nd1	173.4 (14)
O4—Nd1—O1—C1	−60.7 (5)	O11—N5—O12—Nd1	−14 (2)
O3—Cu1—O2—C7	160.0 (4)	O2—Nd1—O12—N5	40.2 (16)
N2—Cu1—O2—C7	100.1 (6)	O3—Nd1—O12—N5	−29.4 (14)
N1—Cu1—O2—C7	−34.0 (4)	O9—Nd1—O12—N5	94.2 (13)
Nd1—Cu1—O2—C7	173.2 (5)	O5—Nd1—O12—N5	−146.2 (13)
O3—Cu1—O2—Nd1	−13.20 (15)	O12'—Nd1—O12—N5	3(5)
N2—Cu1—O2—Nd1	−73.1 (6)	O11—Nd1—O12—N5	7.7 (11)
N1—Cu1—O2—Nd1	152.82 (19)	O6—Nd1—O12—N5	−116.9 (12)
O3—Nd1—O2—C7	−161.7 (4)	O8—Nd1—O12—N5	147.6 (15)
O9—Nd1—O2—C7	56.7 (4)	O11'—Nd1—O12—N5	20.3 (12)
O5—Nd1—O2—C7	−62.4 (4)	O4—Nd1—O12—N5	−77.1 (13)
O12'—Nd1—O2—C7	117.4 (8)	O1—Nd1—O12—N5	135.8 (11)
O11—Nd1—O2—C7	133.4 (6)	O2—Cu1—N1—C8	23.6 (4)
O6—Nd1—O2—C7	−87.7 (4)	O3—Cu1—N1—C8	77.0 (8)
O12—Nd1—O2—C7	108.9 (6)	N2—Cu1—N1—C8	−144.8 (4)
O8—Nd1—O2—C7	24.7 (4)	Nd1—Cu1—N1—C8	44.1 (5)
O11'—Nd1—O2—C7	126.5 (7)	O2—Cu1—N1—C9	−156.4 (4)
O4—Nd1—O2—C7	−155.9 (3)	O3—Cu1—N1—C9	−103.1 (6)
O1—Nd1—O2—C7	−17.7 (3)	N2—Cu1—N1—C9	35.1 (5)
O3—Nd1—O2—Cu1	11.59 (13)	Nd1—Cu1—N1—C9	−135.9 (4)
O9—Nd1—O2—Cu1	−130.06 (19)	O2—Cu1—N2—C13	55.3 (8)
O5—Nd1—O2—Cu1	110.87 (18)	O3—Cu1—N2—C13	−3.3 (4)
O12'—Nd1—O2—Cu1	−69.3 (7)	N1—Cu1—N2—C13	−171.8 (4)
O11—Nd1—O2—Cu1	−53.3 (5)	Nd1—Cu1—N2—C13	−2.2 (5)
O6—Nd1—O2—Cu1	85.59 (17)	O2—Cu1—N2—C12	−123.6 (6)
O12—Nd1—O2—Cu1	−77.8 (6)	O3—Cu1—N2—C12	177.8 (4)
O8—Nd1—O2—Cu1	−162.10 (15)	N1—Cu1—N2—C12	9.2 (4)
O11'—Nd1—O2—Cu1	−60.3 (6)	Nd1—Cu1—N2—C12	178.9 (3)
O4—Nd1—O2—Cu1	17.4 (2)	Nd1—O6—N3—O7	171.1 (6)
O1—Nd1—O2—Cu1	155.6 (2)	Nd1—O6—N3—O5	−8.2 (5)
O2—Cu1—O3—C19	−168.3 (4)	Nd1—O5—N3—O7	−171.0 (6)
N2—Cu1—O3—C19	−2.0 (4)	Nd1—O5—N3—O6	8.3 (5)
N1—Cu1—O3—C19	137.2 (5)	O2—Nd1—N3—O6	−20.8 (3)
Nd1—Cu1—O3—C19	179.2 (4)	O3—Nd1—N3—O6	40.4 (3)
O2—Cu1—O3—Nd1	12.51 (14)	O9—Nd1—N3—O6	−118.0 (3)
N2—Cu1—O3—Nd1	178.82 (15)	O5—Nd1—N3—O6	−171.4 (5)
N1—Cu1—O3—Nd1	−42.0 (6)	O12'—Nd1—N3—O6	144.5 (6)
O2—Nd1—O3—C19	169.5 (4)	O11—Nd1—N3—O6	100.8 (8)
O9—Nd1—O3—C19	−141.1 (3)	O12—Nd1—N3—O6	153.4 (5)
O5—Nd1—O3—C19	42.9 (4)	O8—Nd1—N3—O6	−143.0 (3)
O12'—Nd1—O3—C19	−62.7 (8)	O11'—Nd1—N3—O6	88.5 (14)
O11—Nd1—O3—C19	−86.7 (5)	O4—Nd1—N3—O6	97.9 (3)
O6—Nd1—O3—C19	73.9 (3)	O1—Nd1—N3—O6	−73.6 (3)
O12—Nd1—O3—C19	−56.7 (6)	O2—Nd1—N3—O5	150.6 (3)
O11'—Nd1—O3—C19	−101.7 (6)	O3—Nd1—N3—O5	−148.1 (3)
O4—Nd1—O3—C19	−5.0 (3)	O9—Nd1—N3—O5	53.4 (4)
O1—Nd1—O3—C19	134.8 (3)	O12'—Nd1—N3—O5	−44.1 (6)
O2—Nd1—O3—Cu1	−11.30 (13)	O11—Nd1—N3—O5	−87.7 (8)
O9—Nd1—O3—Cu1	38.1 (2)	O6—Nd1—N3—O5	171.4 (5)
O5—Nd1—O3—Cu1	−137.93 (14)	O12—Nd1—N3—O5	−35.2 (5)
O12'—Nd1—O3—Cu1	116.6 (7)	O8—Nd1—N3—O5	28.4 (3)
O11—Nd1—O3—Cu1	92.5 (4)	O11'—Nd1—N3—O5	−100.0 (14)
O6—Nd1—O3—Cu1	−106.93 (16)	O4—Nd1—N3—O5	−90.7 (3)
O12—Nd1—O3—Cu1	122.5 (5)	O1—Nd1—N3—O5	97.9 (3)
O11'—Nd1—O3—Cu1	77.5 (5)	Nd1—O8—N4—O10	178.7 (5)
O4—Nd1—O3—Cu1	174.18 (19)	Nd1—O8—N4—O9	−1.7 (5)
O1—Nd1—O3—Cu1	−46.00 (18)	Nd1—O9—N4—O10	−178.6 (5)
O2—Nd1—O4—C18	0.3 (4)	Nd1—O9—N4—O8	1.8 (5)
O3—Nd1—O4—C18	6.1 (3)	O2—Nd1—N4—O8	−142.4 (3)
O9—Nd1—O4—C18	120.3 (3)	O3—Nd1—N4—O8	169.7 (3)
O5—Nd1—O4—C18	−128.4 (3)	O9—Nd1—N4—O8	−178.2 (5)
O12'—Nd1—O4—C18	122.3 (6)	O5—Nd1—N4—O8	−8.0 (3)
O11—Nd1—O4—C18	80.7 (6)	O12'—Nd1—N4—O8	90.7 (7)
O6—Nd1—O4—C18	−75.3 (3)	O11—Nd1—N4—O8	125.9 (5)
O12—Nd1—O4—C18	134.0 (5)	O6—Nd1—N4—O8	−46.8 (4)
O8—Nd1—O4—C18	179.8 (3)	O12—Nd1—N4—O8	80.4 (5)
O11'—Nd1—O4—C18	74.6 (7)	O11'—Nd1—N4—O8	139.1 (6)
O1—Nd1—O4—C18	−87.0 (4)	O4—Nd1—N4—O8	58.0 (4)
O2—Nd1—O4—C20	−178.0 (4)	O1—Nd1—N4—O8	−87.8 (3)
O3—Nd1—O4—C20	−172.2 (5)	O2—Nd1—N4—O9	35.8 (3)
O9—Nd1—O4—C20	−58.0 (5)	O3—Nd1—N4—O9	−12.1 (5)
O5—Nd1—O4—C20	53.3 (4)	O5—Nd1—N4—O9	170.1 (3)
O12'—Nd1—O4—C20	−56.0 (6)	O12'—Nd1—N4—O9	−91.2 (7)
O11—Nd1—O4—C20	−97.6 (6)	O11—Nd1—N4—O9	−56.0 (5)
O6—Nd1—O4—C20	106.4 (4)	O6—Nd1—N4—O9	131.4 (3)
O12—Nd1—O4—C20	−44.3 (5)	O12—Nd1—N4—O9	−101.4 (5)
O8—Nd1—O4—C20	1.5 (5)	O8—Nd1—N4—O9	178.2 (5)
O11'—Nd1—O4—C20	−103.7 (8)	O11'—Nd1—N4—O9	−42.7 (6)
O1—Nd1—O4—C20	94.7 (4)	O4—Nd1—N4—O9	−123.8 (3)
O2—Nd1—O5—N3	−38.4 (4)	O1—Nd1—N4—O9	90.4 (3)
O3—Nd1—O5—N3	35.5 (3)	C1—O1—C2—C3	−8.7 (7)
O9—Nd1—O5—N3	−140.8 (3)	Nd1—O1—C2—C3	165.5 (4)
O12'—Nd1—O5—N3	141.8 (6)	C1—O1—C2—C7	170.9 (5)
O11—Nd1—O5—N3	119.5 (7)	Nd1—O1—C2—C7	−14.9 (5)
O6—Nd1—O5—N3	−4.7 (3)	O1—C2—C3—C4	−178.8 (5)
O12—Nd1—O5—N3	147.5 (5)	C7—C2—C3—C4	1.6 (8)
O8—Nd1—O5—N3	−149.3 (3)	C2—C3—C4—C5	0.0 (9)
O11'—Nd1—O5—N3	125.4 (12)	C3—C4—C5—C6	−2.1 (9)
O4—Nd1—O5—N3	79.5 (3)	C4—C5—C6—C7	2.5 (8)
O1—Nd1—O5—N3	−77.3 (3)	C4—C5—C6—C8	177.9 (5)
O2—Nd1—O6—N3	160.6 (3)	Cu1—O2—C7—C6	26.4 (6)
O3—Nd1—O6—N3	−137.9 (3)	Nd1—O2—C7—C6	−161.4 (4)
O9—Nd1—O6—N3	91.0 (4)	Cu1—O2—C7—C2	−154.7 (3)
O5—Nd1—O6—N3	4.8 (3)	Nd1—O2—C7—C2	17.5 (6)
O12'—Nd1—O6—N3	−48.9 (7)	C5—C6—C7—O2	178.0 (5)
O11—Nd1—O6—N3	−109.7 (6)	C8—C6—C7—O2	2.8 (8)
O12—Nd1—O6—N3	−34.7 (5)	C5—C6—C7—C2	−0.9 (7)
O8—Nd1—O6—N3	39.9 (3)	C8—C6—C7—C2	−176.1 (5)
O11'—Nd1—O6—N3	−131.0 (8)	C3—C2—C7—O2	179.9 (5)
O4—Nd1—O6—N3	−73.4 (3)	O1—C2—C7—O2	0.3 (6)
O1—Nd1—O6—N3	99.4 (3)	C3—C2—C7—C6	−1.2 (7)
O2—Nd1—O8—N4	43.6 (3)	O1—C2—C7—C6	179.2 (4)
O9—Nd1—O8—N4	1.0 (3)	C9—N1—C8—C6	174.1 (5)
O5—Nd1—O8—N4	171.4 (3)	Cu1—N1—C8—C6	−5.9 (8)
O12'—Nd1—O8—N4	−81.7 (8)	C7—C6—C8—N1	−13.3 (9)
O11—Nd1—O8—N4	−57.2 (5)	C5—C6—C8—N1	171.4 (5)
O6—Nd1—O8—N4	142.9 (3)	C8—N1—C9—C10	−166.0 (5)
O12—Nd1—O8—N4	−88.3 (6)	Cu1—N1—C9—C10	14.1 (7)
O11'—Nd1—O8—N4	−42.7 (5)	N1—C9—C10—C11	−91.0 (6)
O4—Nd1—O8—N4	−135.9 (3)	C9—C10—C11—C12	49.6 (7)
O1—Nd1—O8—N4	82.8 (3)	C13—N2—C12—C11	98.1 (5)
O2—Nd1—O9—N4	−144.2 (3)	Cu1—N2—C12—C11	−82.9 (5)
O3—Nd1—O9—N4	172.9 (3)	C10—C11—C12—N2	60.2 (6)
O5—Nd1—O9—N4	−11.2 (4)	C12—N2—C13—C14	−175.7 (4)
O12'—Nd1—O9—N4	81.2 (5)	Cu1—N2—C13—C14	5.4 (7)
O11—Nd1—O9—N4	121.8 (5)	N2—C13—C14—C19	−1.6 (7)
O6—Nd1—O9—N4	−71.9 (4)	N2—C13—C14—C15	176.8 (5)
O12—Nd1—O9—N4	69.8 (4)	C19—C14—C15—C16	0.7 (7)
O8—Nd1—O9—N4	−1.0 (3)	C13—C14—C15—C16	−177.8 (4)
O11'—Nd1—O9—N4	133.3 (7)	C14—C15—C16—C17	0.6 (8)
O4—Nd1—O9—N4	83.1 (4)	C15—C16—C17—C18	−1.0 (8)
O1—Nd1—O9—N4	−80.3 (3)	C16—C17—C18—O4	179.8 (5)
O2—Nd1—N5'—O12'	−172.2 (17)	C16—C17—C18—C19	0.1 (8)
O3—Nd1—N5'—O12'	128.3 (18)	C20—O4—C18—C17	−8.2 (7)
O9—Nd1—N5'—O12'	−99.4 (18)	Nd1—O4—C18—C17	173.4 (4)
O5—Nd1—N5'—O12'	11 (2)	C20—O4—C18—C19	171.5 (5)
O11—Nd1—N5'—O12'	143 (3)	Nd1—O4—C18—C19	−6.9 (5)
O6—Nd1—N5'—O12'	79 (2)	Cu1—O3—C19—C14	5.6 (6)
O12—Nd1—N5'—O12'	−7(2)	Nd1—O3—C19—C14	−175.3 (3)
O8—Nd1—N5'—O12'	−52.0 (18)	Cu1—O3—C19—C18	−175.4 (3)
O11'—Nd1—N5'—O12'	176 (3)	Nd1—O3—C19—C18	3.6 (5)
O4—Nd1—N5'—O12'	66.2 (17)	C15—C14—C19—O3	177.4 (4)
O1—Nd1—N5'—O12'	−109.3 (18)	C13—C14—C19—O3	−4.2 (7)
O2—Nd1—N5'—O11'	11.5 (18)	C15—C14—C19—C18	−1.5 (6)
O3—Nd1—N5'—O11'	−48.0 (17)	C13—C14—C19—C18	176.9 (4)
O9—Nd1—N5'—O11'	84.3 (16)	C17—C18—C19—O3	−177.9 (4)
O5—Nd1—N5'—O11'	−165.1 (15)	O4—C18—C19—O3	2.4 (6)
O12'—Nd1—N5'—O11'	−176 (3)	C17—C18—C19—C14	1.2 (7)
O11—Nd1—N5'—O11'	−33 (2)	O4—C18—C19—C14	−178.5 (4)
